# Only lasers can be used for low level laser therapy

**DOI:** 10.1051/bmdcn/2017070422

**Published:** 2017-11-24

**Authors:** Sergey Vladimirovich Moskvin

**Affiliations:** State Scientific Center of Laser Medicine Moscow 121165 Russia

**Keywords:** Low level laser therapy, Monochromaticity, Medicine, Veterinary

## Abstract

The question of lasers' exclusivity, as well as the degree of influence of special properties of low-intensity laser illumination (LILI), such as coherence, polarity and monochromaticity, on the effectiveness of low level laser therapy (LLLT) continues to cause arguments.

The study analyzes publications from 1973 to 2016, in which laser and conventional light sources are compared, and the following conclusions are drawn. First, there are a lot of publications with incorrect comparison or unfounded statements. Secondly, other sources of light are often meant by LILI without any justification. Thirdly, all studies, in which the comparison is carried out correctly and close parameters of the impact and the model are used, have a firm conclusion that laser light is much more effective. Fourthly, it is uniquely identified that the most important parameter that determines the efficiency of lasers is monochromaticity, *i.e.*, a much narrower spectral width than for all other light sources.

Only laser light sources can be used for LLLT!

Translational medicine promotes a faster implementation of scientific achievements in the field of practical public health, allowing a personalization of treatment, which positively affects its results. This interaction was described as “Bench-to-Bedside” or “Bedside-to-Bench” [1]. This is an interdisciplinary field of modern medicine, based on the achievements of science: physiology, molecular biology, genetics and clinical research, created to ensure a higher efficiency of medical services.

Laser therapy is a vivid example of interdisciplinary medicine, which was based on the fundamental research in the field of physiology, biophysics and biochemistry, resulting in the emergence of highly effective therapeutic techniques that take into account the individual characteristics of the patient. However, it is only possible to see the full potential of laser therapy by strictly following the rules, and using appropriate equipment.

## 1. Historical background

Therapeutic properties of “concentrated” light, *i.e.* lamp *(e.g.* UV, blue or red) isolated by narrow part light filter from total spectral irradiation range, were known already in the nineteenth century. This discovery formed the basis for a new field of medicine – light- or phototherapy, and in 1903 N.R. Finsen was awarded the Nobel Prize “in recognition of his contribution to the treatment of diseases, especially lupus vulgaris, with concentrated light radiation, whereby he opened a new avenue for medical science”.[2] All researchers of that time were convinced that to improve effectiveness of phototherapy it was necessary to meet the following conditions: decrease the width of the spectral range to the limit and set optimal light capacity, contacted area, exposure [3-10].

Low level laser therapy–a method of treatment which appeared in the late 1960’s in countries in Eastern Europe, followed by a significant development in Russia [11, 12], and is now continuing to gain recognition around the world. The results of numerous studies of the laws of biomodulating action (BMA) of low-intensity laser illumination (LILI), carried out on animals, and their treatment regimens formed the basis of the method, widely used both in veterinary medicine and medicine: urology, neurology, dentistry, pediatrics, otorhinolaryngology, gynecology, *etc.* [12-19].

One of the problems that hinders the development of low level laser therapy nowadays is the use of other light sources instead of lasers.

The term Low Level Laser Therapy (LLLT) originally came about to be specifically about lasers [20], but more and more often, the abbreviation LLLT was read as “low level laser (light) therapy” [21, 22], or the word “laser” was replaced by “light” as a synonym [23], unequivocally declaring the alleged absence of differences [24] and guided by good intentions, so as not to “get confused” [25]. The motivation for these actions is strange: “Both laser and ordinary light are photons, light is light, so there is no difference” [26, 24].

It is so far unclear, why such statements are made. This is a dangerous assumption, and, for example, you cannot use a jackhammer instead of a scalpel for a surgical operation, despite both tools being made of iron.

## 2. Differences of lasers from other light sources

The main property of laser light is its monochromaticity, there is only one wavelength in spectrum, and this is what determines its higher efficiency unachievable for other light sources.

Laser light is not only monochromatic, but also allows to set and control its energy, allocate it over the surface and deliver to the required location without loss than to do the same with an average lamp with a filter, which were used by N.R. Finsen and his followers. Lasers appeared to be a fundamentally more effective instrument to achieve a therapeutic effect than other light sources, which determined the emergence of a qualitatively new direction of phototherapy–low level laser therapy [1, 12].

Before analyzing literature and comparative studies, it is necessary to understand technical terms and issues. In particular, the comparison of the spectrum of different light sources and their modes of operation.

In many scientific works, the abstract term “coherence” is used without focusing on two separate components of this term: spatial and temporal, which are fundamentally different in terms of their physical meaning and their very core, and therefore, must be treated independently. In short, spatial coherence is the distance at which the light flux remains coherent, and does not affect the efficiency of biomodulation, only because it disappears almost immediately in the upper layers of the skin. But here, the temporal coherence, or the degree of monochromaticity is retained until it is completely absorbed in the biological tissue.

The role of polarisation in BMA is not discussed in detail as that is the subject of as separate study, however, it is worth mentioning that for laser sources, its contribution to the overall result is small, but when using broad spectrum light sources, it is extremely important. Unpolarised light is often completely useless from a medical point of view.

The analysis of various studies, own scientific research, experience and basic knowledge of the fundamentals of biophysics make it easy to state that it is impossible to consider the question of the significance of specific properties of laser light from the point of view of extremes, whether there is absolute “coherence”. It is necessary to estimate the width of the spectral line with specific numbers for the correct interpretation of the experimental data, to allow the change from qualitative estimates to quantitative ones.

Modern techniques to vary the width of the spectral line with the control of the exact value of this index make it possible to successfully carry out experimental work in this field. Oftentimes, we can compare the BMA of laser (or light emitting diode-LED) with thermal or gas-discharge light sources (lamps). The latter, with the help of various monochromators (interference filters, diffraction gratings, *etc.),* a relatively narrow spectral line of a width up to 8-14 nm is cut out at the wavelength of the laser in the comparison study. The incoherent illumination of all light sources, other than lasers, is called “monochromatic incoherency”, “narrow-band light”, “incoherent narrow-band”, *etc.* [27-30].

**Fig. 1:**
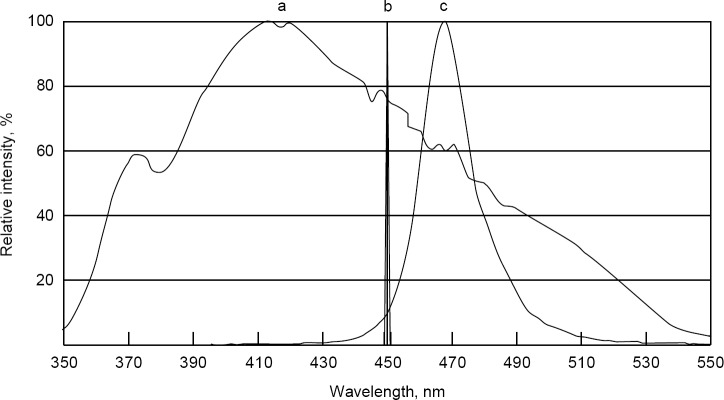
- Spectra of a Finsen lamp with a special light filter (a), a PL 450B OSRAM (b) laser diode and an NHSB046AT NICHIA light emitting diode (c).

Fig. 1 shows the comparison of the spectra of a lamp with a special light filter, a light-emitting diode and a laser diode. The first graph is drawn from the work in which the authors measured the transmission spectrum of devices from the museum N.R. Finsen [31], the typical spectra of LEDs and laser diodes are given from the catalogues of NICHIA and OSRAM, respectively. The LED has a spectrum narrower than the old lamps and they are more convenient to use, but it is in no way comparable to the width of the spectral line of laser diodes, as it is practically one wavelength!

**Fig. 2:**
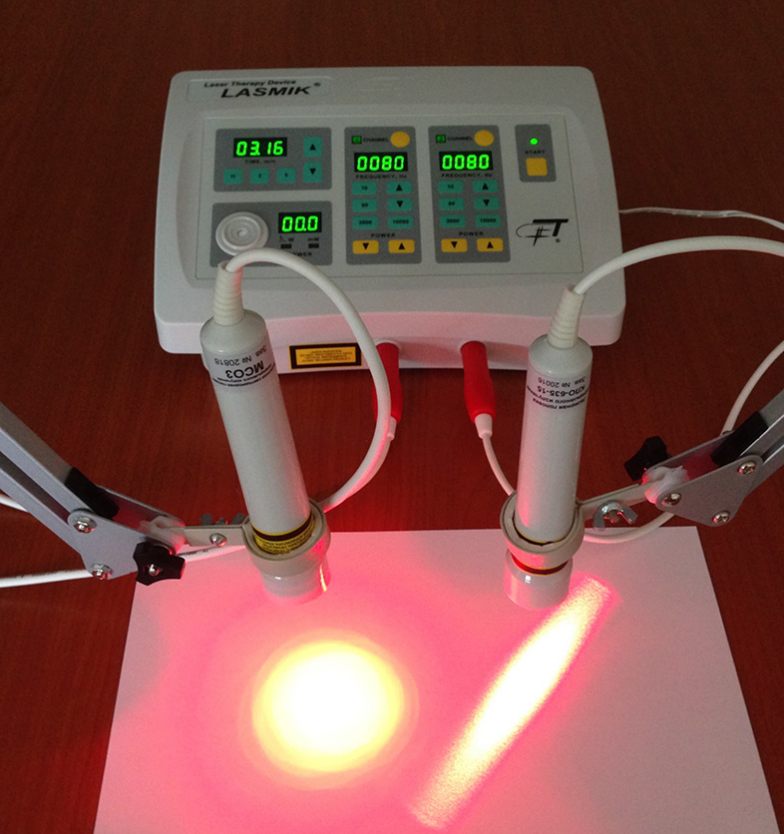
- The light spot of two sources with one wavelength (635nm): LED bottom left (power 60 mW) and laser diodebottom right. (power 15 mW)

Lamps for light therapy are almost not used at present, only LEDs, which differ from the laser diodes (LD) by the width of the spectral line and the light spot - in LEDs it is round and homogeneous, in LD - in the form of an ellipse and has a granular structure (Fig. 2) Both devices with one wavelength are made from one semiconductor material, they are crystals and diodes, but they cannot be distinguished in appearance (by device housing).

In connection with this comparison, it is worth mentioning that this is fraud, and is simply said to be “legitimate” when it is convenient for the “treatment”, a conventional LED lamp is offered without any filter, and it is precisely as its advantage is claimed (http://www.bioptron.com/How-it-Works/Bioptron-Light-Therapy.aspx): “A broad range of wavelengths from 480 to 3400 nm, containing the color range of visible light wavelengths plus a part of the infrared spectrum. Different light wavelengths penetrate the skin at different depths, activating cells, accelerating local blood circulation and stimulating the whole body’s regenerative processes”. We can only sympathize with those who have already spent a lot of money on absolutely useless “treatments”, and advise those wishing to “be treated” in this way, to buy an ordinary table lamp at any hardware store that will cost hundreds of dollars (!) less.

Laser diodes can operate both in continuous mode, including with modulation, and pulsed mode, and LEDs can only work in continuous mode and with modulation, which greatly limits their use.

## 3. Modes of operation of light sources and basic methods of low level laser therapy

Low-intensity laser illumination (LILI) in continuous mode is more often used for laser acupuncture (wavelength 635 nm, power 2-3 mW) and intravenous laser blood illumination (ILBI) (wavelength 365, 405, 445, 525 and 635 nm, power from 2 to 20 mW), and (but less often) for local exposure, when the lesion is localized close to the surface (different wavelengths, power from 10 to 200 mW) [32-34]. Sometimes, incoherent LED light is also used in these techniques, albeit with less efficiency.

Continuous illumination can be modulated, *i.e.,* change its intensity during the procedure, as is done with a signal lamp on ships, switching on/off that which transmits the message with the Morse code. Both laser and conventional light may be modulated, but modulation is used quite rarely and it is often confused with pulsed mode. If there are such things such as pulsed laser diodes, then such LEDs do not exist.

Pulsed lasers do not operate in a continuous mode in practice, but they generate impulses with high pulse power (for therapy use, the power is usually used is from 10 W to 100 W) at a constant duration (100-200 ns). It is always necessary to indicate the repetition frequency of the pulses in the technique for these lasers, since the average power is proportional to the following formulas:

P_average_ = P pulse × F × **τ**, where:

P_average_ - average power,

P_pulse_ - impulse (peak) power,

F is the repetition rate of impulses,

**τ** - duration of pulses (constant value).

When using the formula, a pulsed power of 10-15 W and a frequency of 80-150 Hz (such parameters are most often used for the infrared (904 nm LLLT), the average power will be approximately 0.1 mW, which is 100-1000 times less than the power used for continuous light sources. Therefore, laser light in the pulsed mode is used 100-1000 times more efficiently than continuous mode to procure similar reactions of the biological systems. But impulse LEDs do not exist, therefore, it is impossible to achieve such efficiency.

Therefore, only LLLT in the pulsed mode allows the implementation of techniques, such as:

- non-invasive laser blood illumination (NLBI);
- affect the deep tissues and organs,
- affect to immunocompetent organs;
- affect the nerve nodes;
- transcranial technique.

There are attempts to carry out NLBI with the help of continuous LLLT *(i.e.,* potentially can be achieved with LED light). For example, the “Chinese” version is an intranasal technique in which the localization is motivated by a close arrangement to the surface of the capillaries, even though it is the mediated role of the nervous system [35, 36]. Many times, it has been discussed that illumination of the peripheral vessels cannot be called NLBI, as it is important to only act upon on large blood vessels (veins and arteries) to obtain an adequate response.

In addition, with the endonasal technique, the effect is performed on the hypersensitive neuroendocrine reflex zone and is accompanied by the reflexive excitation of the hypothalamic formations controlling the secretion of biologically active substances participating in various processes: stimulation of uterine contraction, regulation of the circulatory and reproductive systems, control of the production of various hormones (follicle stimulating hormone, estrogens), *etc.* [37-39]. Therefore, this effect is exclusively indirect, and is not associated with direct exposure to blood. Otherwise all would shine on the lips, since there is simply no better access to the capillaries than there (and no effect either). But the endonasal technique is extremely dangerous with unpredictable consequences, especially for women and their fertility. Unfortunately, this technique has been widespread in China.

## 4. Different sources of light for biomedicine, economics

Arguments upon the topic of “lasers or LEDs” have long since gone from being purely scientific discussion, to discussions from an economic perspective. The fact is that numerous creators of “pseudo-lasers” are actively (and quite successfully) trying to sell such products under the brand name of “LLLT”, justifying their actions precisely by the lack of specificity of laser light and its efficiency. For example, a review by H. Chung *et al.* (2012) [40] which included very few and unreliable studies which did not show the effects from laser exposure, generally stated that the prospects for the development of laser therapy are associated with the use of LEDs, which seem to be more effective. But this is not true.

Such statements are simply made by people who aim to have an unfair advantage. If their devices are so effective, then why do they use the term “laser” and use other brands? Why don't they do some research, show the results, call it something suitable, such as “LED therapy”, or, as suggested by R.C.A. Pizzo *et al.* [41], “LEDytherapy”, and develop it in a whole new direction. The answer to this question is obvious: there is a lack of effects produced from incoherent light sources, the insignificance of the effects, and most people understand that the term “LED therapy” will quickly become useless if not “covered” with highly effective laser therapy, while at the same time discrediting the term laser therapy.

For example, a recently published book had a seemingly unambiguous title of “Handbook of Low-Level Laser Therapy”, but in it, there is almost nothing about lasers, only things about LED’s [42]. The first sentence is “Low-level laser (light) therapy (LLLT)”, while simultaneously on the same page but a little lower is written"… do not necessarily need lasers to carry out LLLT… the use of light-emitting diode (LED) arrays is rapidly taking off, and these devices are readily available on online shopping websites and are also sold on late-night television” and then following this, this becomes a book only about LED. So, the authors decided to replace really effective sources of light for laser therapy - lasers, with really ineffective ones, simply due to their availability. This is like saying: why not replace all drugs with water, which is cheaper and more affordable? The authors of the book have refused to discuss this topic, for reasons known only by them.

## 5. Typical errors in conducting of trials

An objective conclusion can only be made when only one parameter - the width of the spectral line of the light sources - is different in one experimental or clinical model. All other variables of the technique must be kept constant. Therefore, when analysing scientific sources, it is necessary to carefully evaluate the correctness of the comparison, paying special attention to the identity and optimality of all parameters of the techniques.

For example, it is completely unclear on what basis G.A. Zalesskaya *et al.* (2013) [43] drew conclusions “…upon the absence of significant differences in the mechanisms of the effects of laser and non-laser illumination,” when only the shift of the haemoglobin dissociation curve was observed after Ultraviolet Blood Illumination (UVBI) (254 nm, 20 minutes, extracorporeally) and NLBI (670 nm, 15 minutes, per cubital vein). At the same time, the difference in methods – in particular, the wavelength - is not considered. It should also be mentioned that the patients underwent complex treatment, because of which these revealed changes could also occur. It would be the same as comparing the effectiveness of LILI with the effectiveness of morning exercises–completely different methods of treatment, but may yield the same result.

There is an even more vivid example of incorrect conclusions, in which the BMA of laser light and LEDs with different wavelengths were compared on the model of stimulating the proliferation of fibroblasts *in vitro.* Statistical analysis - according to the authors - showed a higher proliferation rate in all groups compared to the control group, but the green LED light (570 nm) significantly stimulated cell division more than the red (660 nm) and the infrared (950 nm), suggesting that all LEDs are more effective than laser light [44]. It is absolutely unacceptable to draw such a conclusion when the energy parameters (power, area, power density (PD), energy density (ED) and exposure) differ for LLLT and incoherent light by tens of times! Only three groups with LEDs can be compared more or less correctly in this study, and even with reservations (Table 1).

**Table 1:** - Parameters of the light sources used in the E.M. Vinck *et al.* (2003)

Wavelength, nm	Power, mW	Area, CM^2^	PD, mW/CM^2^	Exposure, secs	ED, J/CM^2^
830 (laser)	40	0.196	204	5	1
570 (LED)	10		0.56	180	0.1
660 (LED)	160	18	8.89	60	0.53
950 (LED)	80		4.44	120	0.53

de Sousa A.P.C. *et al.* (2013) [45] concluded that the light of both LEDs and laser diodes approximately equally stimulate angiogenesis in animals (Wistar rats), however, for LLLT, the least effective or least optimal wavelengths, 660 nm and 790 nm were chosen, or the concentration of light energy in a point, rather than its distribution over the area, which led to a completely unacceptable PD with unreasonably high power. The exposure of 168 seconds and 200 seconds, to put it lightly, is not optimal. A similar error was made in an earlier paper [46].

There are many publications like these that can be given as an example, and it is likely that someone may soon begin comparing the effectiveness of LED with a laser device which isn't even switched on, and then claim that the latter is completely ineffective!

In a study by T.N. Demidova-Rice *et al.* (2007) [47] there is another problem. No difference was found in the wound healing effect (side excision wounds of 10 × 13 mm in BALB/c and SKH1 mice), both from incoherent lamp light (635 ± 15 nm) and He-Ne laser (633 nm, 2 J/cm^2^) [47], and the reason for this being that an incorrect time of 30 minutes was used for the study. With such exposure, which exceeds the maximum permissible level several times, the effect should be absent regardless of the light source, so it is impossible to draw general conclusions. Specialists also know that exposure to illumination of one zone should in no circumstances exceed 300 seconds (5 minutes) [48].

## 6. Analysis of comparative studies

Many researchers have tested the biostimulating properties of LLLT and light from incoherent sources (depolarized with a wide spectrum) in different models, but the results vary considerably. A lot, obviously, depends on the experimental model, however, the general nature of the conclusions drawn speaks in favour of greater monochromaticity efficiency - the narrower the spectrum, the higher the effect at a lower power density.

One of the first comparisons like this was conducted by D. Haina *et al.* (1973) [49]. Effects on experimental wounds (249 Wistar rats) with He-Ne laser light (group 1) and incoherent light with the same wavelength (group 2). In the first group, the growth of the granulation tissue increased by 13% with ED 0.5 J/ cm^2^ and by 22% with a more optimal EP of 1.5 J/cm^2^, while in the second group, the increase did not exceed 10%.

Our comparative evaluation of the quantitative results from several dozen studies has shown that the therapeutic effect causes light with a spectral bandwidth less than Δλ ≈ 15-20 nm, and with a spectral band width of less than 3-5 nm, further narrowing of the spectrum does not lead to an increase in efficiency [50], which was confirmed by the data of other authors [51]. As an example, there are several studies with known widths of the spectral line of light sources, which confirm our findings.

Experiments by V.A. Dubrovskiy *et al.* (1982) [52] showed that the light absorption coefficient of hemolysate, oxyhemoglobin, and catalase does not depend on the spatial coherence and degree of polarization of light. The contribution of temporal coherence (monochromaticity) is much more significant. The illumination of the investigated molecules directly and with the laser light and with the light of the incandescent lamp through light filters showed that the illumination from the helium-neon laser (He- Ne laser) is absorbed several times (depending on the concentration of the object under study) more actively than incoherent light with a larger spectral width. This advantage of LILI is attributed exclusively to the fact that the absorption coefficient of incoherent light, averaged over the width of the emission spectrum of an incandescent lamp (Δλ ≈ 10 nm), is lower than the corresponding coefficient for a laser beam, determined essentially for only one wavelength.

**Fig. 3:**
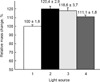
- Influence of the degree of the monochromaticity of the polarized light (power density 2.9mW/cm^2^, exposure 60 seconds), when exposed to fertilized eggs, on the mass of 50-day-old juveniles of sturgeon: 1-control; 2-GNL (Imax = 633 nm, Al = 0.02 nm); 3 - LED (Imax = 631 nm, Al = 15 nm); 4 - “white” LED (Imaxl = 453 nm, AI =¡ 20 nm, Imax2 = 567 nm, AI ~ 130nm). Reproduced with permission of ref [53].

V.U. Plavskii and N.V. Barulin (2009) [53] clearly demonstrated the dependence of the effect on the width of the spectral line with the effect on fertilized eggs of sturgeon. By the way, the third variant, a so-called "white" LED, had wavelengths in another area of the spectrum, specifically, the blue and green (Fig. 3).

Usually, research compares the effect of coherent (lasers) and incoherent light sources. The work of R. Lubart *et al.* (1993) [54] is one of the few that did not include lasers, studied the photobiological effect of light only on LEDs with a wavelength of 540 nm (Δλ ≈ 5nm) and a lamp with a filter in the spectral range of 600-900 nm (Δλ ≈ 300nm), allowing you to draw interesting conclusions. Firstly, we find confirmation of the significance of such a relative index as the spectral power density. The energy parameters optimal for stimulating cell division (human skin fibroblasts) for two different light sources, depend on the power density (upper graphs) and the energy density (lower graphs) with the same exposure (300 seconds) [54]. The effect, albeit insignificant, was observed in both cases, however, the wider the spectrum, the greater (and more significant!) the values of power density and energy are needed to achieve the result. Such conclusion fits perfectly into the model of the thermodynamic triggering of Ca^2+^-dependent processes that we proposed: the narrower the spectrum, the more significant the temperature gradient arising from the absorption of photon energy [55]. In this work, a lot is said about the role of Ca^2+^ in the response to illumination of a living cell.

In experiments with cell culture (mitotic activity of Staphylococcus aureus), almost no differences were found in the effects of LLLI of the single-mode lasers with a spectral line width of less than 0.1 nm and multimode diode lasers with Δλ ≈ 4 nm with a single wavelength (1300 nm) [56]. In this range of values of this index, there are no changes with a decrease in the width of the spectrum, therefore, one should not strive to use single-mode, let alone single-frequency lasers in laser therapy.

T.I. Karu *et al.* (1982) [57] obtained for HeLa cell culture *in vitro* differences in the growth of permeability of cell membranes for H3-thymidine by 20%, and an increase in DNA synthesis by 15% after exposure to GNP illumination and filtered incoherent light from a lamp with a close wavelength and spectral band width ≈ 14 nm. Laser light was, of course, much more effective. In the opinion of the authors, the absence of a more pronounced dependence of the effect on the width of the spectral line is explained by the difference in the rates of creation and relaxation of coherence. The rate of excitation of the molecules ("creation of coherence") is 0.003-0.03s-1 at a power density of the LLLT being in the range of 1-10 mW cm^2^, while the rate of loss of coherence of excitation due to the dephasing of the wave functions of the excited states of molecules in the same conditions were approximately 1011–1012s–1. That is, the significance of the spectral line width in the achieved effect is directly related to the effective absorption cross section of the molecule.

M. Boulton and J. Marshall (1986) [58], observing an increase in the proliferation of fibroblasts *in vitro* alongside a 15-minute previous illumination using He-Ne laser (633 nm) and a halogen lamp with a filter (640 nm, Δλ ≈ 9 nm), showing that if LILI significantly speeds up the process (by 20-40%), then the lamp’s light has no effect. However, the parameters of the method were very strange, the exposure was much larger than the optimal values, the power density was only 0.1 mW cm^2^, and the laser was being operated in modulation mode (F = 100 Hz, τ ≈ 3 ms, Q = 3), which does not ensure absolute correctness for a comparison, as the lamp was working continuously.

S. Rochkind *et al.* (1989) [59] studied the therapeutic efficacy of light at five different wavelengths when exposed to peripheral nerves. The illumination of the He-Ne laser (633 nm) resulted in an increase in the functional activity of the damaged nerve, while incoherent light (660 nm) proved to be much less effective, and the effect of infrared LLLT (830 nm) and incoherent light (880 nm and 950 nm) did not cause any effect.

He-Ne laser stimulates the activity of lymphocytes and macrophages *in vitro,* causes an increase in phagocytic activity, the release of immunoglobulins. A similar result is not observed when exposed to ordinary monochromatic light with the same wavelength (at maximum) and at the same energy density [60, 61].

Reliably better (by 45%) than in the control group and with the use of LEDs, there was wound healing in the group of animals (Wistar rats) when they were exposed to the laser diode illumination (wavelength 830-840 nm, ED was chosen to be optimal, equal to 1 J/cm^2^), that is, the full inefficiency of LEDs is demonstrated on this model [62].

If laser light (He-Ne laser) significantly increases the viability of sea urchins, sea cucumbers and bivalve mollusks, then the LED (850 nm) has no influence [63].

J. Kubota, T. Ohshiro (1989) [64], on the model of artificial injury (Wistar rats) showed that after illumination with a diode laser (830 nm), the bruised tissues had better perfusion, more capillaries, and significantly increased blood flow velocity. These differences in the groups of the rats that were LED illuminated (840 nm) and in the control group were not observed.

P. Pöntinen (1995) [65] measured by the method of laser Doppler flowmetry of the state of capillary blood flow to the scalp of healthy men 30 minutes after the effect of LLLT (670 nm, ED 0.12-0.36 J/cm^2^ per four zones) and LED (635 nm, EP 0.68-1.36 J/cm^2^) showed that laser light leads to an increase in local blood flow, whereas the illumination from LEDs causes a reverse effect.

E.L. Laakso *et al.* (1994) [66] examined 56 patients with chronic pain syndromes according to the double-blind control method, a significant increase in adrenocorticotropic hormone (ACTH) and β-endorphin levels was observed in the two groups of laser therapy (820 nm wavelength, 25 mW power, and 670 nm wavelength, Power 10 mW). The effect was not observed in the group of patients who were illuminated with LEDs (wavelength 660 nm, spectral width 30 nm, power 9.5 mW).

Phototherapy with a lamp for men with the syndrome of delayed muscle pain (660-950 nm, 31.7 J/cm^2^, exposure 12 minutes, modulated, frequencies 2.5, 5 and 20 Hz) was completely ineffective [67]. Here, again, we must make a reservation regarding the non-optimal exposure.

I. Bihari and A. Mester (1989) [68] conducted a comparative evaluation of treatment (with a double-blind control) of three groups of patients with long-healing ulcers of the lower limbs. In the first group, only He-Ne laser was used for illumination, in the second group - He-Ne laser and diode laser, and in the third group - incoherent and unpolarised light. Patients in groups one and two were cured (in group two the results were somewhat better than in the first group), in the third group no significant effects were observed.

Similar regularities were also revealed in experiments with plant cells, if low-intensity laser illumination of the He-Ne laser (633nm) exerts a significant stimulating effect on morphogenetic processes (the formation of zones of secondary differentiation, rhizogenesis, regeneration) in the culture of wheat tissue, then incoherent light with the same wavelength does not cause a cell reaction [69].

One more circumstance is noteworthy. In placebo-controlled studies LEDs often serve as light sources that simulate a laser, but do not have therapeutic effect. For example, it is shown that incoherent light does not exert any influence on patients with heroin addictions with a pronounced effect after laser exposure. [70-72]

## 7. Conclusion

Thus, NON-laser light sources (lamps with or without filters, LEDs with or without a polarizer, *etc.)* cannot be used in low level laser therapy because of their minimal efficiency.

Obviously, LEDs have their niche in a vast field of light therapy, for example, they are very successfully used in photodynamic therapy, UV LEDs have a good bactericidal effect, but to expect clinical effects similar to those obtained precisely in low level laser therapy using laser light (LLLT) from them may be a waste of time.

Laser therapy–as the name suggests, should only be conducted with lasers but no other light sources!

## Conflicts of Interest

The author declares that there is no conflict of interest.
